# Voltammetric Determination of Pb(II) by a Ca-MOF-Modified Carbon Paste Electrode Integrated in a 3D-Printed Device

**DOI:** 10.3390/s20164442

**Published:** 2020-08-09

**Authors:** Evaggelia Vlachou, Antigoni Margariti, Giannis S. Papaefstathiou, Christos Kokkinos

**Affiliations:** 1 Laboratory of Analytical Chemistry, Department of Chemistry, National and Kapodistrian University of Athens, 15771 Athens, Greece; ev412@hotmail.com; 2 Laboratory of Inorganic Chemistry, Department of Chemistry, National and Kapodistrian University of Athens, 15771 Athens, Greece; antmargariti@gmail.com (A.M.); gspapaef@chem.uoa.gr (G.S.P.)

**Keywords:** metal-organic frameworks, 3D-printed electrode, voltammetry, lead

## Abstract

In this work, a voltammetric method based on a metal organic framework (Ca-MOF)-modified carbon paste electrode for lead determination was developed. The MOF-based electrode was packed in a new type of 3D-printed syringe-type integrated device, which was entirely fabricated by a dual extruder 3D printer. After optimization of the operational parameters, a limit of detection of 0.26 µg L^−1^ Pb(II) was achieved, which is lower than that of existing MOF-based lead sensors. The device was used for Pb(II) determination in fish feed and bottled water samples with high accuracy and reliability. The proposed sensor is suitable for on-site analyses and provides a low-cost integrated transducer for the ultrasensitive routine detection of lead in practical applications.

## 1. Introduction

Lead ion is considered one of the most toxic heavy metal ions and is concentrated in aquatic ecosystems and poses a serious hazard to human health, even at trace levels [1,2,3]. Numerous diseases are related to Pb(II), such as anemia, cardiovascular and developmental disorders, and muscle paralysis, while a low concentration of Pb(II) can harm the brain, liver, and nerves [2,3]. Thus, for health and environmental protection, it is vital to employ sensitive and routine methods suitable for on-site monitoring of this hazardous element. Different spectrometric techniques (the most frequently applied is inductively coupled plasma mass spectrometry (ICP-MS)), are used for the measurement of lead concentration, but they require expensive and bulky instrumentation as well as expert technicians, which make them unsuitable for on-site applications [4,5]. On the other hand, anodic stripping voltammetry (ASV), providing inexpensive and portable instrumentation, simple operational protocols, and enhanced analytical sensitivity, has been recognized as the gold standard technique for heavy metal determination [6,7]. Commonly, the ASV determination of lead is carried out for mercury, bismuth, and antimony film electrodes formed either by electrodeposition or the sputtering process [8,9,10].

Nowadays, metal-organic frameworks (MOFs), which are assembled from metal clusters and organic ligands, have gained considerable attention as novel functional materials, thanks to their exceptional features, such as high porosity, plethora of functional groups, and adjustable chemical functionality [10,11,12,13,14,15,16,17,18,19]. However, only a few works have managed to use MOFs as an electrode material or modifier due to their poor electronic conductivity and water stability [13]. Regarding the ASV determination of Pb(II), UiO-66-NH_2_ MOF, [13], MIL-100(Cr) MOF [14], Ni-MOF [15], NH_2_-MIL-53(Cr) MOF [16], and MOF-5 [17] have been applied as electrode modifiers offering limits of detection from 1.5 × 10^−9^ to 5.1 × 10^−7^ mol L^−1^. However, these applications require the use of “large-size” external reference and counter electrodes [13,14,15,16,17], while the fabrication of MOF-based electrodes (except an MOF-5 modifier) follows a multistep procedure based on drop-casting of an MOF on the surface of a glassy carbon electrode (GCE) [13,14,15,16]. According to the drop-casting process, each time and before each electrochemical measurement, the bare GCE surface is abraded and polished, cleaned via sonication in different solutions, and then the MOF is dripped onto the GCE and air-dried. Consequently, these drop-casted MOF-based electrodes cannot be considered ready-to-use and complete sensors.

**Figure 1:**
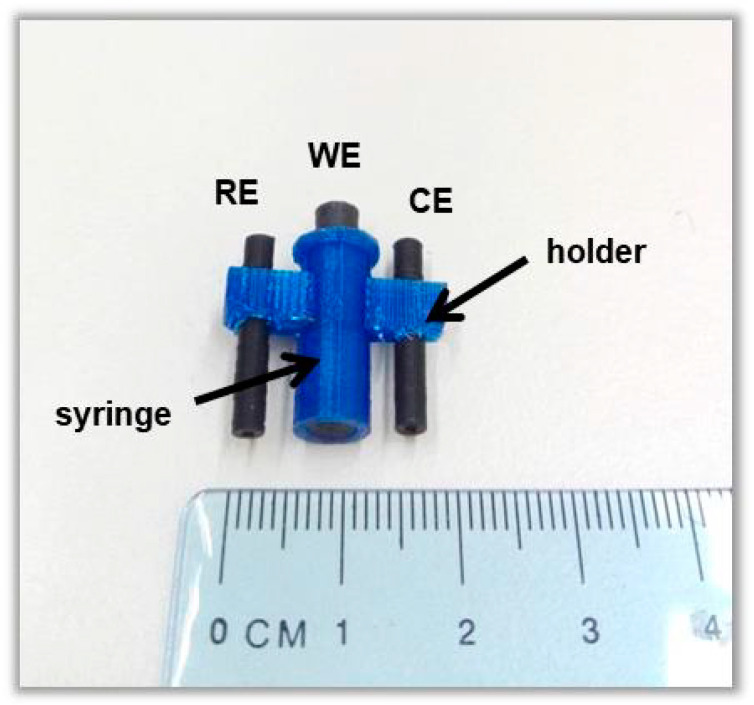
Photograph of the 3D-printed device.

We have recently reported a 2D Ca-MOF ([Ca(H_4_L)(DMA)_2_]·2DMA where H_6_L is the N,N’-bis(2,4-dicarboxyphenyl)-oxalamide and DMA is the *N,N*-dimethylacetamide), which is insoluble in aqueous media and presents sorption and exchange properties towards many heavy metal cations [18,19]. Here, we show that this Ca-MOF, which is a highly efficient Pb(II) sorbent, can be utilized as an electrode modifier by its mixing with graphite paste (GP). The Ca-MOF/GP mixture is packed in a 3D-printed syringe, which serves as the working electrode (WE) of a 3D-printed electrochemical device. The device is fabricated entirely by a dual extruder 3D printer and it also features two conductive electrodes (counter, pseudo-reference) printed from a carbon-loaded polylactic acid (PLA) filament and an electrode holder printed from a PLA non-conductive filament (Figure 1). The electric contact of the WE with the potentiostat is established through a conductive plunger (printed from conductive PLA filament). The WE surface can be renewed by slight pressing on the syringe plunger and removing the excess of the Ca-MOF/GP at the tip of the syringe. The device was used for Pb(II) ASV determination in fish feed and bottled water samples.

As we have shown in our previous studies, the Ca-MOF is capable of exchanging the Ca^2+^ ions with other divalent metal ions, such as Pb(II), Cd(II), Ni(II), Zn(II) or Cu(II), when immersed in their aqueous solutions as evidenced by the absence of Ca^2+^ in the final M-MOFs [M(II) = Pb(II), Cd(II), Ni(II), Zn(II) or Cu(II)] materials [18,19]. Constricting further discussion to Pb(II), the Ca-MOF showed one of the highest Pb(II) sorption capacities (~522 mg g^−1^) reported for MOFs [18]. The nature of the Pb-MOF and the mechanism of the Ca^2+^ exchange by Pb(II) were elucidated by the combination of various spectroscopic and physical techniques. We have found that the Ca-MOF is capable of exchanging its one Ca^2+^ ion (per formula unit) with two Pb(II) cations, a process that involves further deproprotonation of the oxalamide ligand, H_6_L, to afford its tetraanionic form, i.e., H_2_L^4−^, in the exchanged product, Pb-MOF. We have also isolated a new form Pb(II) MOF from the direct reaction of PbCl_2_ with H_6_L, which also supports the presence of two Pb(II) ions per one H_2_L^4−^ ligand. Further evidence of the nature of the Pb-MOF is provided here by highlighting some salient features of the thermogavimetric analysis. The TGA analysis of a Pb-MOF over silica gel dried overnight exhibits a weight loss of 23.17% between room temperature and 225 °C (Figure S1). If we take that the residue at 225 °C (76.83%) as [Pb_2_(H_2_L)], then the Pb-MOF is formulated as Pb_2_(H_2_L)(H_2_O)_14_. With this formula in mind, the removal of 14 H_2_O molecules corresponds to 24.99% (experimental 23.17 %), while the residue of 35.96 above 600 °C corresponds to two Pb (theoretical value 38.4 %). It is worth mentioning that the other M-MOF materials [M(II) = Ni(II), Zn(II) or Cu(II)], obtained by the same method, exhibited lower mass residues at high temperatures (i.e., above 600 °C), which is in accordance with the exchange of one Ca^2+^ by one M(II) [18,19].

The developed MOF-based 3D-printed device presents distinct and significant advantages over existing MOF-based electrodes used for the ASV of Pb(II) [13,14,15,16,17]. The use of 3D-printing technology for the preparation of the device offers plenty of smart features including desktop-sized equipment, very low costs, production speed, strict control of the printing parameters, and ease of printing operation, while it generates negligible non-toxic waste [20,21,22,23,24,25,26,27,28,29,30,31,32]. The adopted process of mixing a small quantity of MOF with GP to produce the WE is by far simpler than drop-casting leading to stand-alone sensors, as the surface of the WE is renewed via a slight pressure on the syringe plunger. Finally, the 3D-printed MOF-based device features integrated counter and reference electrodes (thus they do not require any external electrodes) and is suitable for on-site analysis, while the final geometry of the device fits to any conventional voltammetric cell. For the printing of counter and pseudo-reference electrodes, the same material (carbon-loaded PLA) is chosen for fabrication simplicity and speed. The use of 3D-printed carbon-loaded electrodes as a pseudo-reference electrode has been showed to be adequate for most electrochemical applications [33,34,35].

## 2. Materials and Methods

### 2.1. Reagents and Apparatus

The stock solutions of metal cations were prepared by dilution of 1000 mg L^−1^ atomic absorption standards with double-distilled water. The non-conductive PLA filament was purchased from 3DEdge (Athens, Greece), while the conductive PLA filament was carbon-loaded and was obtained from Proto-Pasta (Vancouver, BC Canada) (both had a diameter of 1.75 mm). A microwave digestion oven (CEM, Mars X, North Carolina, USA) was used for the digestion of the fish feed sample. The electrochemical experiments were carried out with a PGSTAT101 (Metrohm Autolab, Ioannina, Greece) potentiostat, and the baseline correction of the voltammograms was performed with NOVA 1.8 software (Metrohm Autolab, Ioannina, Greece). The dual extruder 3D-printer was a Creator Pro from Flashforge. The Ca-MOF and Pb-MOF were synthesized as described previously [18,19]. Thermogravimetric analyses (TGA) were carried out on a Mettler-Toledo TGA/DSC1, Athens, Greece instrument under a N_2_ flow of 50 mL min^−1^ from room temperature to 800 °C with a heating rate of 10 °C min^−1^.

### 2.2. Fabrication of the 3D-Printed Ca-MOF-Based Device

A photograph of the 3D-printed sensor is presented in Figure 1. The device was designed with Tinkercad software, while Flashprint software was used for the printing process, applying a temperature of the platform: 60 °C, temperature of head dispensers: 200 °C, and printing speed: 70 mm s^−1^. For the fabrication of the graphite paste electrode modified with the Ca-MOF, 0.30 g graphite powder (grade #38, Fisher Scientific, Athens, Greece), 60 mg of Ca-MOF, and 0.10 g paraffin were mixed in a mortar. The resulting paste was transferred to the 3D-printed syringe, and a 3D-printed conductive plunger was used as an electric connector. Similarly, unmodified GP electrodes without Ca-MOF were also fabricated for comparison purposes.

### 2.3. Electrochemical Measurements

All potentials are referred to with respect to the carbon pseudo-RE, and the voltammetric measurements were conducted in the presence of oxygen. The sample was placed in the cell, and preconcentration was conducted at −1.2 V for 360 s under stirring. Afterwards, the WE was subjected to an anodic differential pulse (DP) voltammetric scan (pulse 40 mV for 10 ms, scan rate 10 mV s^−1^), and then the voltammogram was recorded. Quantification was carried out by standard additions of Pb(II). Linear sweep (LS) voltammetry of the 3D-printed MOF-based device was conducted in 0.1 mol L^−1^ acetate buffer (pH 4.5) with a scan rate of 50 mV s^−1^.

### 2.4. Preparation of Samples

The fish feed sample and the bottled water were obtained from a local supermarket. For fish feed preparation a portion of 0.25 g of fish feed was spiked with Pb(II) and was digested in a microwave (350 Watt) for 20 min in the presence of 5 mL HCl (30%). The resulting solution was buffered to pH 4.5 using 0.1 mol L^−1^ ammonia solution and diluted with 0.1 mol L^−1^ acetate buffer (pH 4.5) to a final volume of 50 mL. The final concentration of Pb(II) in the working solution was 7.5 µg L^−1^. The bottled water was acidified with 1.0 mol L^−1^ acetate buffer (pH 4.5) and spiked with Pb(II) to a final concentration of 10 µg L^−1^. For ASV analysis, 5 mL of each sample was added to the voltammetric cell.

## 3. Results and Discussion

### 3.1. Voltammetric Determination of Pb(II)

**Figure 2:**
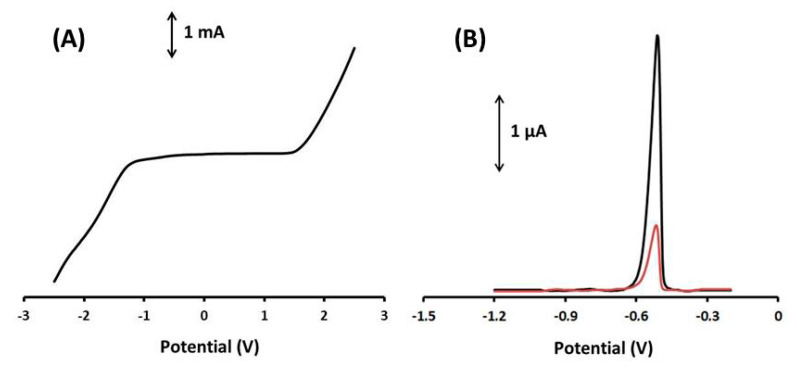
(**A**) LS voltammograms of the 3D-printed Ca-MOF device in 0.1 mol L^−1^ acetate buffer (pH 4.5). Scan rate, 50 mV s^−1^. (**B**) DP voltammogram of 20 µg L^−1^ of Pb(II) (black line) obtained with the modified GPE with Ca-MOF and DP voltammograms of 20 µg L^−1^ of Pb(II) (red line) obtained with the bare GPE in 0.1 mol L^−1^ acetate buffer (pH 4.5).

Firstly, the graphite paste electrode modified with Ca-MOF (Ca-MOF/GP) was tested by LS in 0.1 mol L^−1^ acetate buffer (pH 4.5) at potential range from −2.5 to 2.5 V (Figure 2A). For the voltammogram, the anodic limit was set by the oxidation of water and the cathodic limit by the reduction of hydroxonium ions. The useful operational potential window was about 3V, and as Pb(II) gives an anodic peak at about −0.52 V, the presented electrode is suitable as a Pb sensor. The effective working area of the electrode was determined to be 0.17 cm^2^ using the Randles–Sevcik equation (Figure S2).

In addition, the Ca-MOF/GP sensor was compared to bare GPE in terms of their response to Pb(II) using DPASV (Figure 2B). The DPASV sensitivity of Pb(II) at the Ca-MOF/GP sensor was about four times higher than that of the unmodified graphite paste electrode (GPE), confirming the enhanced voltammetric features of Ca-MOF and the suitability of the device for the voltammetric analysis of Pb(II).

An important aspect of the device is the performance of the 3D-printed carbon pseudo-reference electrode, in terms of both the potential stability and the within-sensor potential reproducibility. The potential stability of the 3D-printed carbon pseudo-reference electrode was studied for 20 repetitive measurement, while the within-sensor potential reproducibility was measured at five different 3D-printed carbon pseudo-reference electrodes. All comparative measurements were conducted in a solution containing 50 µg L^−1^ Pb(II) in 0.1 mol L^−1^ acetate buffer (pH 4.5) (Figure S3). The potential, in which the Pb voltammetric peak appears, remained statistically stable during the 20 repetitive measurements, while the % relative standard deviation (%RSD) of the potential recorded at five different 3D-printed carbon pseudo-reference electrodes was 2.4%. These data indicate that the potential stability of the 3D-printed carbon pseudo-reference electrode and the within-electrode potential reproducibility are satisfactory.

**Figure 3:**
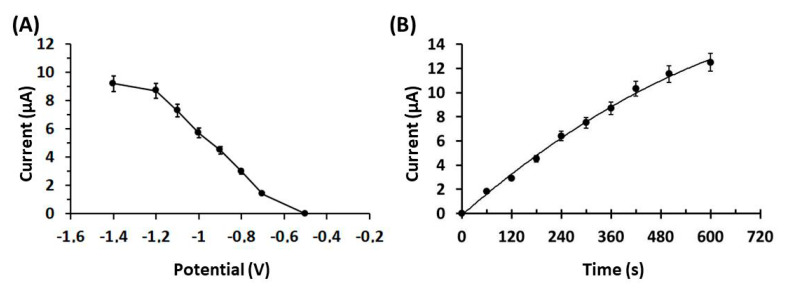
(**A**) Effect of the preconcentration potential (with respect to the carbon pseudo-RE) on the peak heights of 50 µg L^−1^ Pb(II) in 0.1 mol L^−1^ acetate buffer (pH 4.5) at the Ca-MOF/GP. (**B**) Effect of the preconcentration time of 50 µg L^−1^ Pb(II) in 0.1 mol L^−1^ acetate buffer (pH 4.5) at Ca-MOF/GP. The points in the plots are the mean value ± SD (*n* = 4). Other DPASV parameters were pulse 40 mV for 10 ms and scan rate 10 mV s^−1^.

To enhance the electroanalytical performance of the 3D-printed Ca-MOF device for lead determination, the effect of the preconcentration potential and time of the response of 50 µg L^−1^ of Pb(II) in 0.1 mol L^−1^ acetate buffer (pH 4.5) were optimized (Figure 3). As depicted in Figure 3A, the peak current of Pb(II) was high and almost constant at potential values from −1.4 to −1.2 V, and then gradually decreased at more positive potentials. A preconcentation potential of −1.2 V (with respect to the carbon pseudo-RE) was chosen for further experiments.

Regarding the effect of the electrolytic preconcentration time on the DPASV sensitivity of Pb(II), it was tested in the range of 0–600 s (Figure 3B). The peak current increased in a rectilinear fashion with respect to the preconcentration time: at lower preconcentration times, the peak current increased rapidly (from 60–360 s), while at longer preconcentration times (higher than 360 s) the rate of increase dropped. A preconcentration time of 360 s was selected for further experiments, as an optimum compromise between high sensitivity and fast analysis.

**Figure 4:**
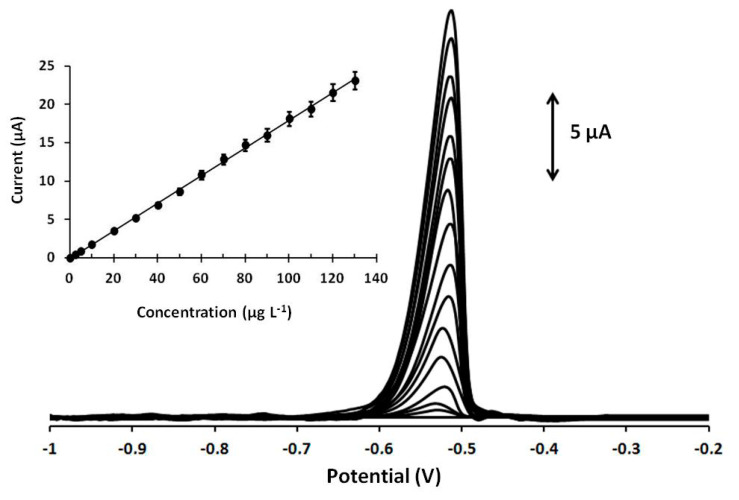
DP voltammograms at the Ca-MOF/GPE for increasing concentrations of Pb(II) in the range of 0–130 µg L^−1^ (0, 2.5, 5, and 10–130 with a 10 µg L^−1^ step of Pb(II)). The corresponding calibration plot is shown as an inset. Each bar is the mean value ± sd (*n* = 3). Supporting electrolyte: 0.1 mol L^−1^ acetate buffer (pH 4.5); preconcentration potential: −1.2 V; preconcentration time: 360 s. Other DPASV parameters were pulse 40 mV for 10 ms and scan rate 10 mV s^−1^.

Calibration of Pb(II) at various concentrations was performed at the Ca-MOF sensor, and the device showed a linear concentration dependence in the tested concentration range [0–130 µg L^−1^ for Pb(II)] ( Figure 4). The calibration curve is expressed by the following first-order equation:

I_Pb_ (μA) = (0.18 ± 0.001)[Pb(ΙI)] (μg L^−1^) + (0.06 ± 0.09), r^2^ = 0.998 (1)

The limit of detection (LOD) was 0.26 µg L^−1^ (1.25 10^−9^ mol L^−1^) and was calculated using the equation LOD = 3 sd/a, where sd is the standard deviation of the intercept of the calibration plot and a is the slope of the calibration plot. This *LOD* is lower than that obtained with existing MOFs used for Pb(II) determination [13,14,15,16,17]. More specifically, the LOD of the presented Ca-MOF 3D-printed device is significantly lower than that offered by MIL-100(Cr) [14], Ni-MOF [15], NH_2_-MIL-53(Cr) [16], and MOF-5 [17] (ranged from 4.9 10^−9^ to 5.1 10^−7^ mol L^−1^) and slightly lower than that obtained with a drop-casted UiO-66-NH_2_ MOF-based electrode in which the LOD is 1.5 10^−9^ mol L^−1^ [13]. The repeatability of the Ca-MOF-based electrode was studied by testing 30 µg L^−1^ Pb(ΙΙ) for five repeated measurements, and the RSD% was calculated at 4.2%. In addition, three different 3D-printed sensors of Ca-MOF/GPEs were prepared for the determination of 30 µg L^−1^ Pb(ΙΙ), and the RSD% of the measurements for the three sensors was 5.8% (*n* = 3). These results confirmed satisfactory reproducibility of the device. The stability of the Ca-MOF/GPE was tested over a period of three months, and a *t*-test confirmed that the electrochemical response of the device was statistically unaffected.

**Figure 5:**
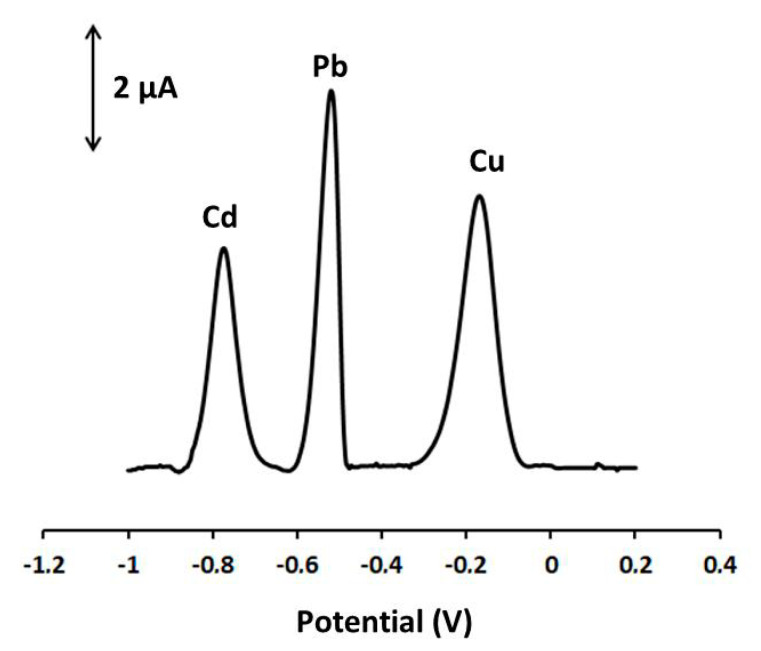
DP voltammograms at the Ca-MOF/GPE of 40 µg L^−1^ Pb(II), 80 µg L^−1^ Cd(II), and 80 µg L^−1^ Cu(II) in 0.1 mol L^−1^ acetate buffer (pH 4.5). Preconcentration potential: −1.2 V; preconcentration time: 360 s; pulse 40 mV for 10 ms; scan rate: 10 mV s^−1^.

Cu(II), Cd(II) may interfere with the determination of Pb(II), but at the Ca-MOF/GPE in 0.1 mol L^−1^ acetate buffer (pH 4.5), Cu(II) and Cd(II) presented peaks that were well separated from the respective Pb peak (Figure 5). Indeed, Cu(II) and Cd(II) did not significantly interfere with the analysis of Pb(II) at 10-fold higher concentrations over Pb(II) (signal variation lower than 15% in the case of Cd and lower than 10% in the case of Cu). Besides, in the presence of other common cations, such as Mn(II), Co(II), Ni(I), Na(I), K(I), Ca(II), Mg(II), Zn(II) at a 10-fold excess over Pb(II), the voltammetric signal of Pb remained almost unaffected (signal variation lower than 5%).

### 3.2. Applications

**Figure 6:**
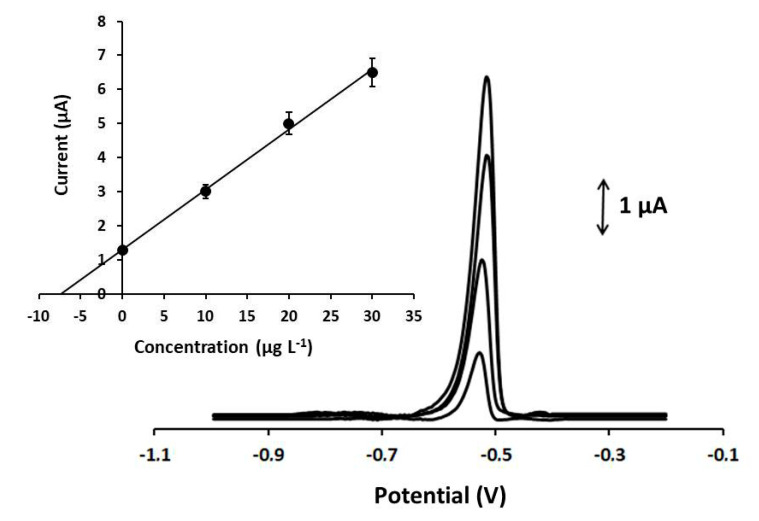
DP voltammograms for the determination of Pb(II) in spiked fish feed sample with 7.5 µg L^−1^ of Pb(II) at the Ca-MOF/GPE. From below: spiked sample and three standard additions of 10 µg L^−1^ of Pb. (Inset) Standard additions curve. Supporting electrolyte: 0.1 mol L^−1^ acetate buffer (pH 4.5); preconcentration potential: −1.2 V; preconcentration time: 360 s; pulse 40 mV for 10 ms; scan rate: 10 mV s^−1^.

To test the practicality of the method, the 3D-printed Ca-MOF device was applied to the determination of lead concentration in spiked fish feed and bottled water samples, following the procedure described in Section 2.4. Both analyses were conducted by the standard addition method, and the representative DP voltammograms of Pb(II) in the fish feed sample are presented in Figure 6. In addition, the samples were analyzed by electrothermal atomic absorption spectrometry (ETAAS, Perkin Elmer Analyst 600, Akron, OH, USA) and the values are listed in Table 1. The electrochemical 3D-printed MOF sensor exhibited adequate accuracy with a relative error of about 4% and recoveries between 97% and 102%. These results indicate that the device can be successfully used as a sensor for the sensitive and accurate analysis of Pb(II) in complex samples.

## 4. Conclusions

In this study, a 3D-printed syringe-type voltammetric sensor for lead determination is described that features a Ca-MOF-modified carbon paste working electrode. Under optimized conditions, the sensor allowed a limit of detection for lead at the sub μg L^−1^ level and satisfactory precision. Applications in detecting Pb(II) in fish feed and bottled water samples also demonstrated that the presented device can be used for analyses of real samples with high accuracy and reliability. The favourable electroanalytical performance, the easy renewal of the electrode surface as well as the scope for rapid and low-cost 3D-printed fabrication make these sensors very promising for on-site monitoring purposes.

## Figures and Tables

**Table 1:** Recovery of Pb(II) in Spiked Samples and Comparison with ETAAS.

Sample	Pb Content (μg L^−1^) ^a^	Pb Added (μg L^−1^)	Pb Determined (μg L^−1^) ^b^	Recovery (%)	Pb Determined with ETAAS (μg L^−1^) ^b^	Relative Error (%)
Fish oil	<LOD	7.5	7.3 (± 0.5)	97	7.6 (± 0.3)	−3.9
Bottled water	<LOD	10	10.2 (± 0.7)	102	9.8 (± 0.4)	4.1

^a^ Measured with ASV and ETAAS; ^b^ Mean value ± sd (*n* = 3).

## Supplementary Materials

The following are available online at https://www.mdpi.com/1424-8220/20/16/4442/s1, TGA curve, Determination of the active area of the electrode, Potential stability of the 3D-printed carbon pseudo-reference electrode.
