# Supplementary material for: Voltammetric Determination of Pb(II) by a Ca-MOF-Modified Carbon Paste Electrode Integrated in a 3D-Printed Device
Source: Sensors (Basel). 2020 Aug 9;20(16):4442. doi: 10.3390/s20164442 (PMC7472020; doi:10.3390/s20164442)
Supplement: Supplementary file 1 [file sensors-20-04442-s001.pdf]

Supplementary Material

Voltammetric Determination of Pb(II) by a Ca-MOF-Modified Carbon Paste Electrode Integrated in a 3D-Printed Device

Evaggelia Vlachou [1], Antigoni Margariti [2], Giannis S. Papaefstathiou [2] and Christos Kokkinos [1,*]

[1]Laboratory of Analytical Chemistry, Department of Chemistry, National and Kapodistrian University of Athens, Athens, 157 71, Greece
[2]Laboratory of Inorganic Chemistry, Department of Chemistry, National and Kapodistrian University of Athens, Athens, 157 71, Greece
*Corresponding author. Email: christok@chem.uoa.gr; Tel.: +302107274312

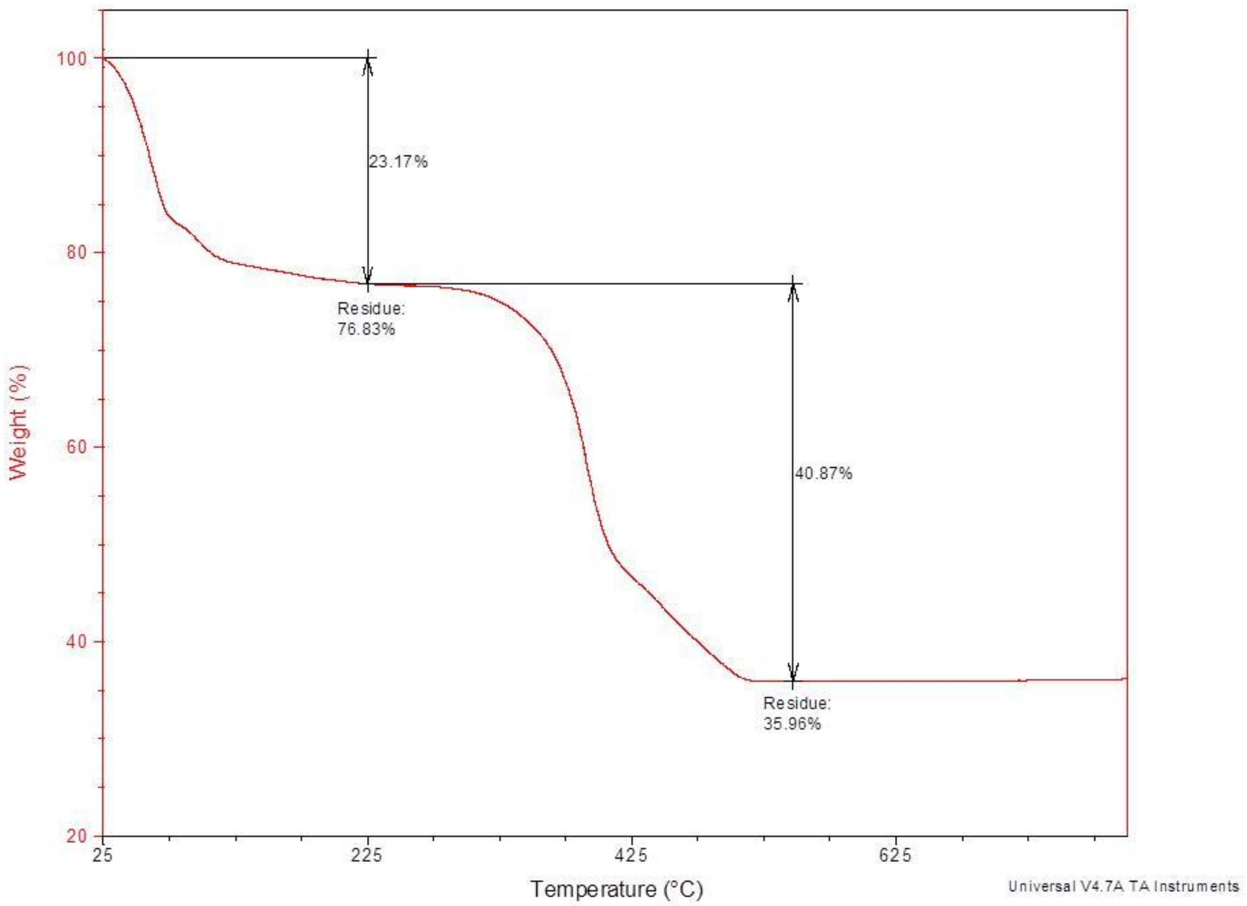

Figure S1. TGA curve of Pb-MOF obtained by immersing Ca-MOF in a $PbCl_2$ aqueous solution for 30 min, followed by filtration and drying over silica gel in a desiccator overnight.

Determination of the Active Area of the Electrode

The effective working area of the electrode was determined using cyclic voltammetric conditions (scan rate 0.05 V s^{-1}) for the one-electron reduction of $K_3[Fe(CN)_6]$ [1.0 mmol L^{-1} in 0.5 mol L^{-1} KCl (Figure S2)] using the Randles-Sevcik equation.

$$i_p = 0.446nFAC(nFDv/RT)^{1/2}$$

where i_p is the peak current (A), n (=1) is the number of electrons transferred, A is the effective area of the electrode(cm^2), D is the diffusion coefficient of $[Fe(CN)_6]^{3-}$ (taken to be $7.60 \times 10^{-6} cm^2 s^{-1}$), C is the concentration (mol cm^{-3}), v is the scan rate (Vs^{-1}), F is the Faraday constant (C mol^{-1}) R is the universal gas constant, T is the temperature in Kelvin.

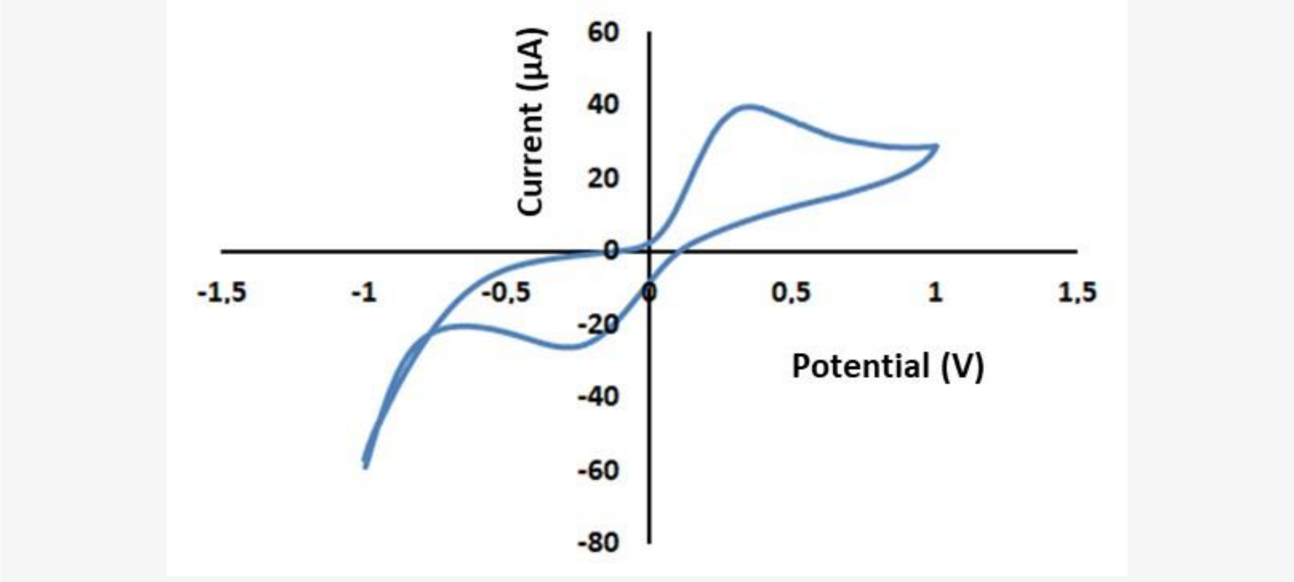

Figure S2. Cyclic voltammogram of $K_3[Fe(CN)_6]$ (1.0 mmol L^{-1} in 0.5 mol L^{-1} KCl) at Ca-MOF/GP. Scan rate: 0.05 V s^{-1}

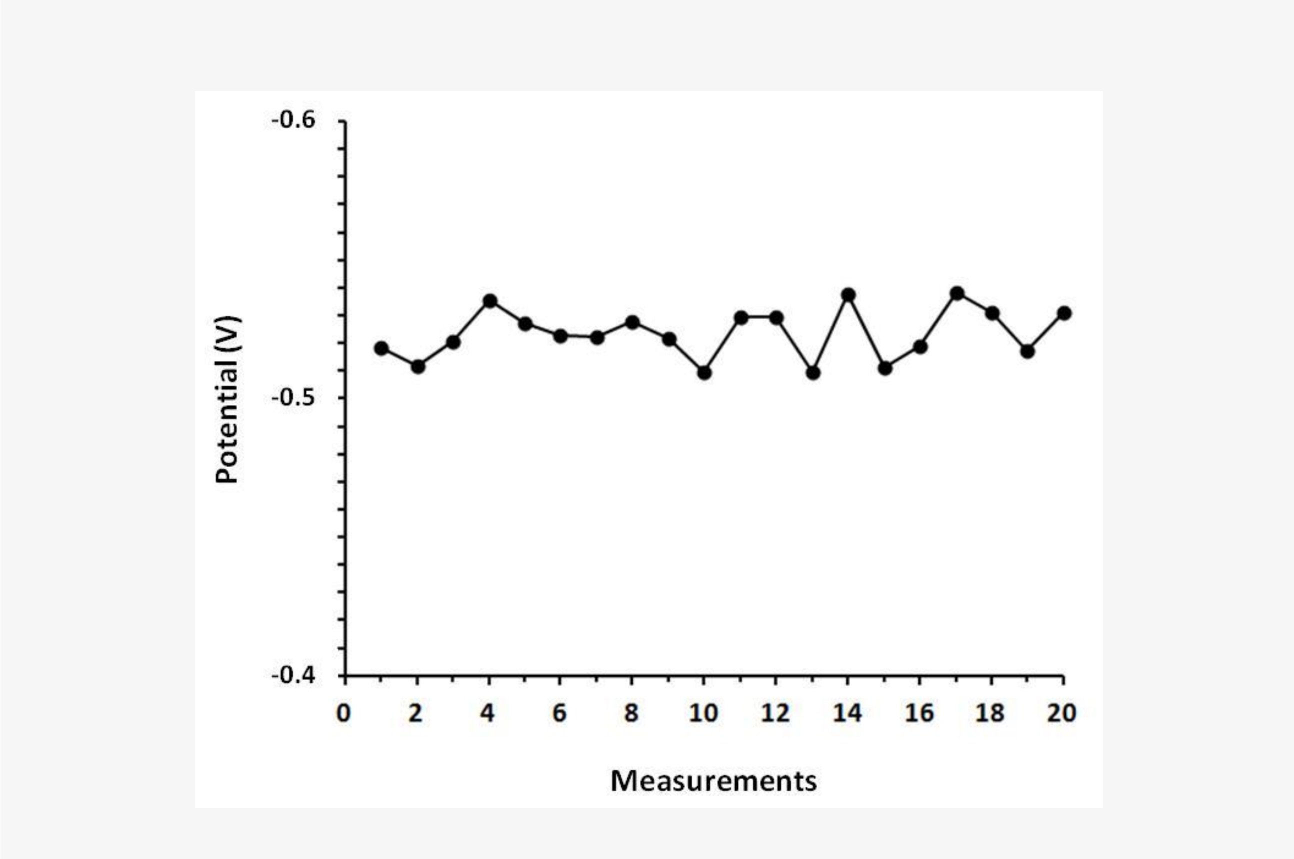

Figure S3. Potential stability of the 3D-printed carbon pseudo-reference electrode. The measurements were conducted in a solution containing 50 µg L^{-1} Pb(II) in 0.1 mol L^{-1} acetate buffer (pH 4.5)
